# Associations of Substance Use Behaviors With Suicidal Ideation and Suicide Attempts Among US and Chinese Adolescents

**DOI:** 10.3389/fpsyt.2020.611579

**Published:** 2021-01-18

**Authors:** Lan Guo, Wanxin Wang, Xueying Du, Yangfeng Guo, Wenyan Li, Meijun Zhao, Ruipeng Wu, Ciyong Lu

**Affiliations:** ^1^Department of Medical Statistics and Epidemiology, School of Public Health, Sun Yat-sen University, Guangzhou, China; ^2^Guangdong Provincial Key Laboratory of Food, Nutrition and Health, Sun Yat-sen University, Guangzhou, China; ^3^Guangdong Engineering Technology Research Center of Nutrition Translation, Guangzhou, China; ^4^Health Promotion Centre for Primary and Secondary Schools of Guangzhou Municipality, Guangzhou, China

**Keywords:** Chinese adolescents, US adolescents, substance use behavior, suicidal behavior, comparison

## Abstract

**Background:** Adolescence has been described as a period of increased health risk-taking behaviors. Given the variety of cultural contexts, healthcare systems, and public health policies in different regions, the present study aimed to determine whether there are similar or different associations of substance use behaviors with suicidal ideation and suicide attempts among US and Chinese adolescents.

**Methods:** This study included a total of 14,765 US adolescents from the 2017 National Youth Risk Behavior Surveillance System (YRBSS) and 24,345 Chinese adolescents from the 2017 School-based Chinese Adolescents Health Survey (SCAHS).

**Results:** The proportions of suicidal ideation and suicide attempts were 17.4 and 5.7% among US adolescents, which were higher than those among Chinese adolescents (suicidal ideation: 13.7% and suicide attempts: 2.7%). Among Chinese adolescents, the most common substance use behavior was “alcohol use (55.4%),” followed by “cigarette use (11.6%).” Among US adolescents, the most popular substance was alcohol (ever used: 55.9%), followed by marijuana (ever used: 34.6%). Moreover, alcohol use was significantly related to suicidal ideation/suicide attempts only in Chinese adolescents [suicidal ideation: Adjusted odds ratio (AOR) = 1.88, 95% CI = 1.71~2.06; suicide attempts: AOR = 2.12, 95% CI = 1.71~2.63], and marijuana use was associated with suicidal ideation and suicide attempts only in the US adolescent group (suicidal ideation: AOR = 1.23, 95% CI = 1.06~1.44; suicide attempts: AOR = 1.51, 95% CI = 1.21~1.87). Moreover, although the associations of prescription pain medication use with suicide attempts were significant in both Chinese and US adolescent groups, the adjusted associations were stronger in Chinese adolescents than in US adolescents (Chinese adolescents: AOR = 3.97, 95% CI = 2.76~5.72; US adolescents: AOR = 1.76, 95% CI = 1.43~2.16; *P* < 0.05).

**Conclusions:** The associations of alcohol use with suicidal ideation and suicide attempts were only significant in Chinese adolescents. Marijuana use was associated with suicidal ideation and suicide attempts only in the US adolescent group. Although the associations of prescription pain medication use with suicide attempts were significant in both Chinese and US adolescent groups, the adjusted associations were significantly stronger for Chinese adolescents. These findings might be related to the differences in cultural contexts, healthcare systems, and public health policies in the two different countries.

## Introduction

Adolescence, defined by the World Health Organization (WHO) as 10–19 years, is a period of immense behavioral, psychological, and social changes. It is also characterized as a stage of elevated exploration and imitation with a range of health risk-taking behaviors (1). Although there is no globally uniform definition of health risk-taking behavior, it is generally considered as behaviors that have negative influences on the development of adolescents, such as suicidal behavior and substance use behavior. Suicidal behavior, including suicidal ideation, suicide attempts, or suicide deaths, has been a substantial public health problem causing nearly one million deaths worldwide in a given year (2), and suicidal ideation and suicide attempts were vital risk factors for death by suicide in late adolescence (3). Evidence suggests that suicidal ideation and attempts are still serious problems among adolescents in developing and developed countries. A previous study using data from the 2017 Youth Risk Behavior Surveillance System (YRBSS) showed that about 17.7% of adolescents had suicidal ideation, and 6.5% attempted suicide during the past 12 months (4). The Centers for Disease Control (CDC) of the United States, using data from the 2019 YRBSS reported that 18.8% of the adolescents had a history of suicidal ideation, and 8.9% attempted suicide at least one time in the preceding 12 months (5). These findings were slightly higher than those described in our prior study among Chinese adolescents, which demonstrated that 16.1% of the students reported having a history of suicidal ideation, and 3.1% reported having attempted suicide (6).

Although suicidal behavior is polyfactorial, substance use behaviors are frequently considered one of the most common risk factors for suicidal behavior (7, 8). According to international drug control conventions, psychoactive substances have clinical significance in strict and reasonable medical use but can be harmful when misused or used for non-medical purposes (9). Among adolescents, cigarette smoking, alcohol consumption, and psychoactive and illicit drug use are common substance use behaviors in modern society (10). However, these behaviors should be prevented as they can inhibit an individual's growth and maturation. There have been several national survey studies regarding adolescent substance use behaviors worldwide. The United States has carried out a project called the YRBSS since 1990. The 2017 YRBSS results report that cigarette use, alcohol use, and illicit drug use are the leading causes of morbidity and mortality during adolescence and later in life. Psychoactive drugs now make up a more significant part of the overall US drug problem than they did 10–15 years ago (11). Although there is no national surveillance system in China to supervise adolescent health risk-taking behaviors, our prior study using data from the SCAHS suggested that substance use behavior among Chinese adolescents is also becoming a significant public health concern (12).

Although the associations of substance use behaviors with suicidal ideation and suicide attempts among adolescents have been well-established, current knowledge surrounding these associations is mostly derived from Western countries (13, 14), with only a few studies undertaken in Chinese adolescents (6, 15). However, it is considered that the variety of cultural contexts, healthcare systems, and public health policies in different regions (16), there is little research on comparing the associations between adolescents from two different cultural and ethnic backgrounds. It is reported that the United States focuses on individualism; China's ideology emphasizes collectivism at the same time (17). Moreover, the traditional intrinsic socio-cultural values and Confucianism (emphasizing family system, education, hierarchical relationships, and benevolence) have influenced China for thousands of years (18), which may make Chinese adolescents differ from their peers in Western countries on the associations of substance use behaviors with suicidal ideation and suicide attempts. Therefore, this study aimed to investigate the associations of substance use behaviors with suicidal ideation and suicide attempts among US and Chinese adolescents and determine whether the associations are similar or different for the two adolescent groups.

## Methods

### Study Design and Participants

Data of the US adolescents were drawn from the 2017 YRBSS, which used a three-stage cluster sample design to produce a representative sample of 9 through 12-grade students from regular public, Catholic, and other private schools in the US. One hundred and ninety-two schools were sampled, and the data of a total of 14,765 US adolescents were downloaded and included in the analyses (19, 20). Data of the Chinese adolescents were drawn from the 2017 SCAHS, which utilized a multi-stage, stratified cluster, random sampling method to produce a representative sample of 7 through 12-grade students from general high schools and vocational high schools in China. Eighty-four schools were sampled, and the data of 24,345 Chinese adolescents were included in the analyses.

### Measures

#### Suicidal Ideation and Suicide Attempts

In the YRBSS and SCAHS, suicidal ideation was assessed with the following question: “during the last 12 months, did you ever seriously consider attempting suicide (yes=1 and no=2)?” Suicide attempts were measured by the question: “during the past 12 months, how many times did you actually attempt suicide?” Available responses were categorized into “yes=1” and “no=2”.

#### Substance Use Behaviors

Information about substance use was also collected, including cigarette use, alcohol use, marijuana use, methamphetamine use, ecstasy use, and prescription pain medication use. Cigarette use was measured by the question: “have you ever tried cigarette smoking, even one or two puffs? (available responses were categorized into yes=1 and no=2)”. Alcohol use was assessed by the following question: “during your life, on how many days have you had at least one drink of alcohol? (available responses were categorized into yes=1 and no=2)”. Marijuana use was assessed by the question: “during your life, how many times have you used marijuana? (responses were categorized into yes=1 and no=2).” Methamphetamine use was measured by the question: “during your life, how many times have you used methamphetamines (also called speed, crystal, crank, or ice) (available responses were categorized into yes=1 and no=2)?” Ecstasy use was measured by the following question: “during your life, how many times have you used ecstasy (also called MDMA) (responses were categorized into yes=1 and no=2)?” Nonmedical use of prescription pain medication was assessed by the following question: “during your life, how many times have you taken prescription pain medication (e.g., codeine, Vicodin, and Percocet) without a doctor's prescription (available responses were also categorized into yes=1 and no=2)?”

#### Other Information

In the YRBSS and SCAHS, bullying at school was evaluated by asking students the question: “during the past 12 months, have you ever been bullied on school property? (available responses were coded as yes=1 and no=2).” Feeling sad or hopeless was measured by the question: “during the past 12 months, did you ever feel so sad or hopeless almost every day for two weeks or more in a row that you stopped doing some usual activities? (responses were coded as yes=1 and no=2).” Information on sociodemographic variables was also collected, including gender (1=female, 2=male) and age (1=12 years old or younger, 2=13 to 17 years old, 3=18 years old or older).

#### Ethical Standards

The authors assert that all procedures contributing to this work comply with the ethical standards of the relevant national and institutional committees on human experimentation and with the Helsinki Declaration of 1975, as revised in 2008. The YRBSS was reviewed and approved by the Centers for Disease Control and Prevention's (CDC) Institutional Review Board. In the YRBSS, student participation was anonymous and voluntary, and local procedures were followed to obtain parental permission (21). The SCAHS was approved by the Sun Yat-sen University School of Public Health Institutional Review Board. In the SCAHS, written informed consent was obtained from each participant who was at least 18 years of age. If the participant was aged under 18 years, the written informed consent was obtained from one of the participant's parents (or legal guardians) (22).

#### Statistical Analysis

Descriptive analyses were used to describe sample characteristics, and chi-square tests were performed to compare the differences between the US and Chinese adolescents. Considering both the YRBSS and SCAHS utilized the multi-stage sampling design in which students were clustered into schools, multi-level logistic regression models were fitted in which schools were treated as groups. Multivariable multi-level logistic regression models were first performed to compare the risk for suicidal ideation and suicide attempts between the US and Chinese adolescents. Moreover, stratified analyses were performed to investigate the factors associated with suicidal ideation and suicide attempts in the US and Chinese adolescent groups, and the statistical significance of the differences between the strata was tested by using the 95% confidence interval (CI):$\;(\beta_{1}\;-\;\beta_{2})\pm 1.96\sqrt{{\left(SE_{1}\right)}^{2}+{\left(SE_{2}\right)}^{2}}$. β_1_ and β_2_ represented the regression coefficients in each stratum, and *SE*_1_ and *SE*_2_ were the corresponding standard errors. The Bonferroni correction for multiple comparisons was also utilized (23). Regarding the logistic regression analyses, observations with missing data were eliminated. All statistical analyses were conducted using Stata SE 12.0 (StataCorp, Houston, Texas, USA), and two-tailed *P* values less than 0.05 were considered statistically significant.

## Results

### Characteristics of the US and Chinese Adolescents

Among the total participants, the proportion of males was 48.2% among US adolescents and 51.5% among Chinese adolescents. Most US and Chinese adolescents were 13 to 17 years old (86.9% among US adolescents and 81.2% among Chinese adolescents). The proportion of suicidal ideation among US and Chinese adolescents was 17.4 and 13.7%, respectively. The proportion rate of suicide attempts among US adolescents was 5.7%, which was higher than that among Chinese adolescents (2.7%). The proportion of US adolescents who reported being bullied at school was 18.0%, and the proportion among Chinese adolescents was 46.5%. The proportion of participants who reported feeling sad or hopeless among US and Chinese adolescents was 31.4 and 41.2%, respectively. Regarding substance use, the proportions of cigarette use, alcohol use, marijuana use, methamphetamine use, ecstasy use, and prescription pain medication use among US adolescents were higher than those among Chinese adolescents. Additionally, significant differences emerged among US and Chinese adolescents in the distribution of gender, age, being bullied at school, feeling sad or hopeless, cigarette use, alcohol use, marijuana use, methamphetamine use, ecstasy use, prescription pain medication use, suicidal ideation, and suicide attempts (*P* < 0.001) (Table 1).

**Table 1:** Characteristics of the Chinese and US adolescents.

**Variable**	**Chinese Adolescents (*N* = 24,345)**	**US Adolescents (*N* = 14,765)**	*P*-value^*^
**Gender**			
Female	11732 (48.2)	7526 (51.0)	<0.001
Male	12526 (51.5)	7112 (48.2)	
Missing data	87 (0.4)	127 (0.9)	
**Age**			
12 years old or younger	1907 (7.8)	59 (0.4)	<0.001
13 to 17 years old	19769 (81.2)	12829 (86.9)	
18 years old or older	2608 (10.7)	1796 (12.2)	
Missing data	61 (0.3)	81 (0.5)	
**Bullying at school**			
Yes	11323 (46.5)	2665 (18.0)	<0.001
No	11934 (49.0)	11941 (80.9)	
Missing data	1088 (4.5)	159 (1.1)	
**Feeling sad or hopeless**			
Yes	10019 (41.2)	4631 (31.4)	<0.001
No	14127 (58.0)	9896 (67.0)	
Missing data	199 (0.8)	238 (1.6)	
**Suicidal ideation**			
Yes	3340 (13.7)	2571 (17.4)	<0.001
No	20033 (82.3)	11982 (81.2)	
Missing data	972 (4.0)	212 (1.4)	
**Suicide attempts**			
Yes	662 (2.7)	837 (5.7)	<0.001
No	23683 (97.3)	9849 (66.7)	
Missing data		4079 (27.6)	
**Cigarette use**			
Yes	2818 (11.6)	3316 (22.5)	<0.001
No	21134 (86.8)	8596 (58.2)	
Missing data	393 (1.6)	2853 (19.3)	
**Alcohol use**			
Yes	13481 (55.4)	8251 (55.9)	<0.001
No	10509 (43.2)	5528 (37.4)	
Missing data	355 (1.5)	986 (6.7)	
**Marijuana use**			
Yes	120 (0.5)	5102 (34.6)	<0.001
No	23965 (98.4)	9180 (62.2)	
Missing data	260 (1.1)	483 (3.3)	
**Methamphetamine use**			
Yes	111 (0.5)	384 (2.6)	<0.001
No	24114 (99.1)	13994 (94.8)	
Missing data	120 (0.5)	387 (2.6)	
**Ecstasy use**			
Yes	115 (0.5)	590 (4.0)	<0.001
No	24147 (99.2)	13761 (93.2)	
Missing data	83 (0.3)	414 (2.8)	
**Prescription pain medication use**			
Yes	323 (1.3)	2051 (13.9)	<0.001
No	23967 (98.4)	12459 (84.4)	
Missing data	55 (0.2)	255 (1.7)	

* *The chi-square test was used for categorical variables*.

### Factors Associated With Suicidal Ideation and Suicide Attempts Among US and Chinese Adolescents

Both among US and Chinese adolescents, females were at a higher risk of suicidal ideation and suicide attempts [Adjusted odds ratio (AOR) = 1.65, 95% CI = 1.53~1.77, and AOR = 1.56, 95% CI = 1.37~1.78]. Students who were bullied at school were more likely to be involved in suicidal ideation (AOR = 2.01, 95% CI = 1.87~2.17) and suicide attempts (AOR = 2.16, 95% CI = 1.89~2.47). Adolescents who felt sad were also at a higher risk of suicidal ideation (AOR = 5.61, 95% CI = 5.20~6.06) and suicide attempts (AOR = 5.98, 95% CI = 5.12~6.98). Students who reported cigarette use, alcohol use, and prescription pain medication use were at a higher risk for suicidal ideation and suicide attempts. Moreover, marijuana use (AOR = 1.23, 95% CI = 1.01~1.50) and methamphetamine use (AOR = 2.31, 95% CI = 1.50~3.54) was only positively associated with suicide attempts. Additionally, compared with Chinese adolescents, US adolescents only had a higher risk of suicide attempts (AOR = 1.78, 95% CI = 1.32~2.41) (Table 2).

**Table 2:** Factors associated with suicidal ideation and suicide attempts among US and Chinese adolescents.

**Variable**	Suicidal ideation^*^		Suicide attempts^*^	
	**AOR (95% CI)**	***P*-value**	**AOR (95% CI)**	***P*-value**
**Gender**				
Male	1.00 (reference)		1.00 (reference)	
Female	1.65 (1.53~1.77)	<0.001	1.56 (1.37~1.78)	<0.001
**Age**				
18 years old or older	1.00 (reference)		1.00 (reference)	
12 years old or younger	1.44 (1.17~1.77)	0.002	0.98 (0.67~1.43)	0.922
13 to 17 years old	1.20 (1.07~1.36)	0.003	0.93 (0.75~1.16)	0.567
**Bullying at school**				
No	1.00 (reference)		1.00 (reference)	
Yes	2.01 (1.87~2.17)	<0.001	2.16 (1.89~2.47)	<0.001
**Feeling sad or hopeless**				
No	1.00 (reference)		1.00 (reference)	
Yes	5.61 (5.20~6.06)	<0.001	5.98 (5.12~6.98)	<0.001
**Cigarette use**				
No	1.00 (reference)		1.00 (reference)	
Yes	1.45 (1.32~1.59)	<0.001	1.90 (1.63~2.22)	<0.001
**Alcohol use**				
No	1.00 (reference)		1.00 (reference)	
Yes	1.65 (1.52~1.78)	<0.001	1.63 (1.39~1.90)	<0.001
**Marijuana use**				
No	1.00 (reference)		1.00 (reference)	
Yes	0.99 (0.87~1.13)	0.919	1.23 (1.01~1.50)	0.014
**Methamphetamine use**				
No	1.00 (reference)		1.00 (reference)	
Yes	1.40 (0.96~2.03)	0.085	2.31 (1.50~3.54)	<0.001
**Ecstasy use**				
No	1.00 (reference)		1.00 (reference)	
Yes	1.08 (0.79~1.47)	0.631	1.23 (0.86~1.77)	0.266
**Prescription pain medication use**				
No	1.00 (reference)		1.00 (reference)	
Yes	1.77 (1.54~2.03)	<0.001	2.01 (1.68~2.42)	<0.001
**Nationality**				
Chinese adolescents	1.00 (reference)		1.00 (reference)	
US adolescents	1.12 (0.95~1.31)	0.149	1.78 (1.32~2.41)	<0.001

*AOR, adjusted odds ratio; 95% CI, 95% confidence interval*.

* *The multivariable logistic regression models for suicidal ideation and suicide attempts incorporating gender, age, bullying at school, feeling sad or hopeless, cigarette use, alcohol use, marijuana use, methamphetamine use, ecstasy use, prescription pain medication use, and nationality*.

### Comparing the Associations of Substance Use Behaviors With Suicidal Ideation and Suicide Attempts Between the US and Chinese Adolescents

As shown in Table 3 and Table 4, alcohol use was significantly related to suicidal ideation and suicide attempts only in Chinese adolescents (suicidal ideation: AOR = 1.88, 95% CI = 1.71~2.06; suicide attempts: AOR = 2.12, 95% CI = 1.71~2.63), and marijuana use was associated with suicidal ideation and suicide attempts only in US adolescent group (suicidal ideation: AOR = 1.23, 95% CI = 1.06~1.44; suicide attempts: AOR = 1.51, 95% CI = 1.21~1.87). The association between cigarette use and suicidal ideation (AOR = 1.70, 95% CI = 1.50~1.92) was significant only in Chinese adolescents, and the associations between cigarette use and suicide attempts were significant in both Chinese (AOR = 2.57, 95% CI = 2.07~3.19) and US adolescent groups (AOR = 1.50, 95% CI = 1.21~1.85). Although the associations of prescription pain medication use with suicide attempts were significant in both Chinese and US adolescent groups, the magnitudes of the adjusted associations were significantly higher in Chinese adolescents than in US adolescents (Chinese group: AOR = 3.97, 95% CI = 2.76~5.72; US group: AOR = 1.76, 95% CI = 1.43~2.16; *P* <0.05).

**Table 3:** Comparing the associations with suicidal ideation between the US and Chinese adolescents.

**Variable**	Suicidal ideation^*^					
	**US adolescents**		**Chinese adolescents**		***P*-value^#^**	***Adjusted P-value^∧^***
	**AOR (95% CI)**	***P-*value**	**AOR (95% CI)**	***P-*value**		
**Gender**
Male	1.00 (reference)		1.00 (reference)			
Female	1.31 (1.15~1.49)	<0.001	1.88 (1.72~2.05)	<0.001	<0.0001	<0.05
**Age**
18 years old or older	1.00 (reference)		1.00 (reference)			
13 to 17 years old	0.81 (0.11~5.92)	0.840	1.35 (1.17~1.55)	<0.001	0.47	5.17
12 years old or younger	1.11 (0.92~1.33)	0.288	1.70 (1.39~2.07)	<0.001	0.10	1.1
**Bullying at school**
No	1.00 (reference)		1.00 (reference)			
Yes	2.23 (1.94~2.56)	<0.001	1.95 (1.79~2.13)	<0.001	0.11	1.21
**Feeling sad or hopeless**
No	1.00 (reference)		1.00 (reference)			
Yes	11.01 (9.58~12.66)	<0.001	4.07 (3.72~4.46)	<0.001	0	0
**Cigarette use**
No	1.00 (reference)		1.00 (reference)			
Yes	1.15 (0.99~1.34)	0.082	1.70 (1.50~1.92)	<0.001	0.0001	0.0011
**Alcohol use**
No	1.00 (reference)		1.00 (reference)			
Yes	1.08 (0.92~1.26)	0.362	1.88 (1.71~2.06)	<0.001	<0.0001	<0.05
**Marijuana use**
No	1.00 (reference)		1.00 (reference)			
Yes	1.23 (1.06~1.44)	0.010	0.63 (0.32~1.24)	0.210	0.06	0.66
**Methamphetamine use**
No	1.00 (reference)		1.00 (reference)			
Yes	1.29 (0.83~2.01)	0.271	1.72 (0.38~7.78)	0.506	0.72	7.92
**Ecstasy use**
No	1.00 (reference)		1.00 (reference)			
Yes	1.10 (0.78~1.55)	0.573	0.91 (0.20~4.09)	0.908	0.81	8.91
**Prescription pain medication use**
No	1.00 (reference)		1.00 (reference)			
Yes	1.71 (1.45~2.02)	<0.001	2.14 (1.62~2.82)	<0.001	0.17	1.87

*AOR, adjusted odds ratio; 95% CI, 95% confidence interval*.

* *The multivariable logistic regression models for suicidal ideation and suicide attempts incorporating gender, age, bullying at school, feeling sad or hopeless, cigarette use, alcohol use, marijuana use, methamphetamine use, ecstasy use, and prescription pain medication use*.

# *The statistical significance of the differences between the US and Chinese adolescents was tested by using the 95% confidence interval, and the observed P-values were reported*.

∧ *The adjusted P-values after Bonferroni correction for multiple testing*.

**Table 4:** Comparing the associations with suicide attempts between the US and Chinese adolescents.

**Variable**	Suicide attempts^*^					
	**US adolescents**		**Chinese adolescents**		***P*-value^#^**	**Adjusted P-value^∧^**
	**AOR (95% CI)**	***P* value**	**AOR (95% CI)**	***P*-value**		
**Gender**
Male	1.00 (reference)		1.00 (reference)			
Female	1.36 (1.13~1.65)	0.003	1.90 (1.58~2.29)	<0.001	0.015	0.165
**Age**
18 years old or older	1.00 (reference)		1.00 (reference)			
13 to 17 years old	1.19 (0.92~1.54)	0.195	1.59 (1.15~2.22)	0.014	0.032	0.352
12 years old or younger	17.98 (2.01~160.50)	0.012	2.38 (1.55~3.65)	0.001	0.007	0.077
**Bullying at school**
No	1.00 (reference)		1.00 (reference)			
Yes	2.34 (1.95~2.80)	<0.001	2.25 (1.85~2.73)	<0.001	0.774	8.514
**Feeling sad or hopeless**
No	1.00 (reference)		1.00 (reference)			
Yes	9.85 (7.78~12.46)	<0.001	3.58 (2.92~4.39)	<0.001	<0.0001	<0.05
**Cigarette use**
No	1.00 (reference)		1.00 (reference)			
Yes	1.50 (1.21~1.85)	<0.001	2.57 (2.07~3.19)	<0.001	0.0005	0.0055
**Alcohol use**
No	1.00 (reference)		1.00 (reference)			
Yes	0.97 (0.76~1.23)	0.779	2.12 (1.71~2.63)	<0.001	<0.0001	<0.05
**Marijuana use**
No	1.00 (reference)		1.00 (reference)			
Yes	1.51 (1.21~1.87)	<0.001	1.78 (0.64~4.98)	0.298	0.754	8.294
**Methamphetamine use**
No	1.00 (reference)		1.00 (reference)			
Yes	1.87 (1.16~3.01)	0.014	20.61 (1.92~220.88)	0.041	0.052	0.572
**Ecstasy use**
No	1.00 (reference)		1.00 (reference)			
Yes	1.26 (0.86~1.86)	0.244	0.16 (0.01~1.86)	0.186	0.103	1.133
**Prescription pain medication use**
No	1.00 (reference)		1.00 (reference)			
Yes	1.76 (1.43~2.16)	<0.001	3.97 (2.76~5.72)	<0.001	0.0001	0.0011

*AOR, adjusted odds ratio; 95% CI, 95% confidence interval*.

* *The multivariable logistic regression models for suicidal ideation and suicide attempts incorporating gender, age, bullying at school, feeling sad or hopeless, cigarette use, alcohol use, marijuana use, methamphetamine use, ecstasy use, and prescription pain medication use*.

# *The statistical significance of the differences between the US and Chinese adolescents was tested by using the 95% confidence interval, and the observed P-values were reported*.

∧ *The adjusted P-values after Bonferroni correction for multiple testing*.

## Discussion

This study, using data from the 2017 YRBSS and 2017 SCAHS, sought to investigate the associations of substance use behaviors with suicidal ideation and suicide attempts among US and Chinese adolescents and determine whether the associations are similar or different for adolescents from two different countries (i.e., the United States and China). The study results revealed that among Chinese adolescents, the most common substance use behavior was “alcohol use (55.4%),” followed by “cigarette use (11.6%),” “prescription pain medication use (1.3%),” and “marijuana/methamphetamine/ ecstasy use (0.5%).” Among US adolescents, the most popular substance was alcohol (ever used: 55.9%), followed by marijuana (ever used: 34.6%), cigarette (ever used, 22.5%), prescription pain medication (ever used: 13.9%), ecstasy (ever used: 4.0%), and methamphetamine (ever used: 2.6%). Moreover, the proportions of students who reported substance use (including cigarette use, alcohol use, marijuana use, methamphetamine use, ecstasy use, and prescription pain medication use) were higher in the US adolescent group than in Chinese adolescent group (*P* <0.001). These results may be related to the cultural values and socialization practices (24), and previous evidence has suggested that culture plays a central role in forming individuals' cognitions about the potential problems they may face with substance use (25). More specifically, it has been suggested that alcohol drinking is a worldwide phenomenon, which is closely associated with many social activities (such as ceremonies and festivals) (26). In this study, alcohol use accounted for more than fifty percent of both the US and the Chinese adolescent group, reflecting that although alcohol's hazards have gradually been recognized by the Chinese and US public, it is clear that more preventive policies and programs need to be accomplished. A prior review demonstrated that China's current public health systems could not monitor or respond well to alcohol drinking and alcohol-related problems (27). Regarding drug misuse, China is thought of as a country that has been rigorous in controlling its drug problems. China has carried out a nationwide campaign to eliminate widespread drug trafficking and abuse according to strictly punishing people involved in growing, transporting, and/or trafficking illicit drugs since the early 1950s (28). In the present study, the observed proportion rate of marijuana/ methamphetamine/ ecstasy/ prescription pain medication use in the Chinese adolescent group was far below that in the US adolescent group. It is notable that although the illicit drug use (e.g., marijuana, methamphetamine, or ecstasy use surveyed in this study) among Chinese adolescents is less common (29), non-medical use of prescription medications has become more prevalent (6). This finding indicates that although the traditional Chinese style of parenting and schooling focuses on strict discipline for misbehavior, global financial and political development may have influenced Chinese adolescents' cultural environment. Moreover, with marijuana being widely legalized for recreational and medicinal purposes in the United States, marijuana use is widespread among US adolescents (30), resulting in the observed higher proportion rate of marijuana use among US adolescents in the present study. Regardless of the differences in culture or the economic, geographical, and political context, it appears that substance use behaviors are common among adolescents in both US and China in contemporary society, making prevention of adolescent substance use a public health priority.

Moreover, this study found that the proportions of suicidal ideation and suicide attempts were respectively 17.4 and 5.7% among US adolescents, which were higher than those among Chinese adolescents (suicidal ideation: 13.7% and suicide attempts: 2.7%); these results were consistent with previous studies (31, 32). In line with prior studies (32), we found that in females, being bullied, feeling sad, cigarette use, alcohol use, and prescription pain medication use were positively associated with suicidal ideation and suicide attempts. These findings suggest that we should mainly focus on those students who present with the aforementioned negative characteristics. Moreover, this study showed that, compared with Chinese adolescents, US adolescents had 1.78 times higher risk of suicide attempts. One possible explanation for this finding might be that due to the influence of traditional Confucian culture (e.g., interpersonal obligations and particularistic role duties) (33), Chinese adolescents may be hesitant or reluctant to disclose their suicidal behavior for the influences of social desirability or parental monitoring (34).

Further stratified analyses were performed to compare the associations of substance use behaviors with suicidal ideation and suicide attempts in the US and Chinese adolescents. The study findings showed that alcohol use was significantly related to suicidal ideation and suicide attempts only in Chinese adolescents, and marijuana use was associated with suicidal ideation and suicide attempts only in the US adolescent group. Moreover, although the associations of prescription pain medication use with suicidal ideation and suicide attempts were significant in both Chinese and US adolescent groups, the magnitudes of the adjusted associations were significantly higher in Chinese adolescents than those in US adolescents. Similarly, previous studies have also identified the associations between substance use and suicidal behavior (8, 13, 14, 35, 36). These results might be related to the idea that substance use may lead to suicidal behavior via impaired judgment, impulsiveness, or loss of inhibition (37).

Moreover, the differences in the associations between substance use and suicidal behavior in the two adolescent groups may be attributed to the different types of prevalent substances. In the present study, it appears that drinking or smoking is more common than other substance use behavior among adolescents in China, and marijuana use was more prevalent in the US adolescent group. Regarding the only significant associations of marijuana use with suicidal behaviors observed in US adolescent group, one potential explanation might be that a smaller sample size of Chinese adolescents reporting marijuana use could not have enough power to yield significant findings. Regarding the higher magnitudes of associations between alcohol/prescription pain medication/cigarette use and suicidal behavior observed in the Chinese adolescent group, a possible explanation from the cultural framework may be that US adolescents may consider this substance use as a personal decision; however, Chinese adolescents were more likely than US adolescents to be influenced by substance use behaviors and to consider these substance uses to be more wrong or even resulting in having suicidal ideation or taking suicide attempts (38). Another explanation based on social epidemiological theories posits that an individual's suicide risk depends not only on their traits and experiences but also on the interplay between social, economic, and environmental factors (39). Although some adolescent suicide prevention interventions (e.g., school-based interventions) have been shown to reduce suicidal behaviors among adolescents in high-income countries effectively, there is a lack of evidence on effective policies or interventions to reduce youth suicidal behaviors from low- and middle-income countries (40).

Based on our study results, we suggest that effective prevention and intervention programs should be established, and the role of the government and social settings should be considered. First, the World Health Organization has proposed that health promotions focused on achieving equality in health and the government's role were crucial for public health (41). The United States government has established several national systems (e.g., YRBSS and Monitoring the Future). A proper surveillance system is suggested to be developed by the Chinese government to monitor and control substance use and suicidal behaviors among adolescents in the long term. Second, family and school roles were well-known factors associated with adolescent health risk-taking behaviors. Along these lines, parents and schools should focus on substance use and suicidal behaviors among adolescents, particularly among those who struggle with family-related or school-related relationship difficulties (42). Third, these study findings may provide preliminary evidence about the importance of focusing on different prevalent substances among US and Chinese adolescents. Early prevention and intervention strategies are recommended to promote resilience among adolescents involved in substance use behaviors to prevent suicidal behavior. For example, school-based intervention programs (including screening, gatekeeper training, peer leadership training, and skills training) may help decrease suicidal behaviors (43, 44). Fourth, to strengthen regulations to supervise adolescents' access to substances (e.g., limiting the sales of alcohol or marijuana to adolescents) may help reduce risky health behaviors (including substance use and suicidal behaviors) among adolescents.

Several limitations of the present study should be noted. First, both YRBSS and SCAHS utilized a school-based design and only included school students. Those adolescents who had dropped out of school were not incorporated; however, substance use behaviors or suicidal behaviors, which are both cause and consequence of their lack of success in school, maybe more common among them (45, 46). A community-based design that can invite adolescents who have dropped out of school is needed in our future study. Second, due to the cross-sectional nature of the YRBSS and SCAHS data, no causal inference can be derived from the associations reported in the paper. The associations need to be further assessed in longitudinal/prospective studies. Third, both YRBSS and SCAHS used the structured self-report questionnaire to collect data. The recall bias cannot be ruled out and may lead to underestimating substance use or suicidal behavior due to social desirability. However, anonymous and voluntary participation is assured in the YRBSS and SCAHS, and this method may have helped in collecting accurate information. Despite these limitations, this study provides evidence of the cross-cultural differences in adolescent substance use and suicidal behaviors. Also, it lays the groundwork for future studies to investigate the mechanism of the differences in substance use and suicidal behaviors between the US and Chinese adolescents.

## Conclusion

In conclusion, the present study highlighted that alcohol drinking and cigarette use are more common than other substance use behavior among Chinese adolescents. After alcohol, the second most popular type of substance was marijuana in the US adolescent group. The stratified results also showed that alcohol use was significantly related to suicidal ideation and suicide attempts only in Chinese adolescents, and marijuana use was associated with suicidal ideation and suicide attempts only in the US adolescent group. Although the associations of prescription pain medication use with suicide attempts were significant in both Chinese and US adolescent groups, the magnitudes of the adjusted associations were significantly higher in Chinese adolescents. These findings suggest that effective prevention and intervention programs are highly recommended to improve the awareness of the adverse effects of substance use and suicidal behavior among adolescents, their families, and schools; to promote resilience among adolescents who have been involved in substance use and suicidal behaviors; to strengthen the active surveillance of these health risk-taking behaviors among adolescents.

## Data Availability Statement

The datasets generated for this study can be found in online repositories. The names of the repository/repositories and accession number(s) can be found at: Data for the SCAHS in China are available through the Sun Yat-sen University. Contact Ciyong Lu for access approval. Data for the YRBSS in the United States are public and are available on the website: https://www.cdc.gov/healthyyouth/data/yrbs/data.htm.

## Ethics Statement

The YRBSS study involving human participants was reviewed and approved by the Centers for Disease Control and Prevention's (CDC) Institutional Review Board; student participation is anonymous and voluntary, and local procedures were followed to obtain parental permission. The SCAHS study involving human participants was reviewed and approved by this study received ethical approval from the Sun Yat-sen University School of Public Health Institutional Review Board; written informed consent to participate in this study was provided by the participants' legal guardian/next of kin.

## Author Contributions

LG and CL had full access to all of the data in the study and take responsibility for the integrity of the data and the accuracy of the data analysis. LG, WW, and CL: concept, design, and drafting of the manuscript. YG, WW, WL, MZ, and RW: statistical analysis. LG and CL: administrative, technical, or material support. LG and CL: supervision. All authors: critical revision of the manuscript for important intellectual content.

## Conflict of Interest

The authors declare that the research was conducted in the absence of any commercial or financial relationships that could be construed as a potential conflict of interest.
